# Clinical and radiographic outcomes following salvage intervention for ureteropelvic junction obstruction

**DOI:** 10.1590/S1677-5538.IBJU.2021.0303

**Published:** 2021-08-01

**Authors:** Joseph J. Crivelli, Brett A. Johnson, Ryan L. Steinberg, Jeffrey C. Gahan, Jodi A. Antonelli, Allen F. Morey, Margaret S. Pearle, Jeffrey A. Cadeddu

**Affiliations:** 1 University of Texas Southwestern Medical Center Department of Urology DallasTX United States Department of Urology, University of Texas Southwestern Medical Center, Dallas, TX, United States;; 2 University of Texas Southwestern Medical Center Internal Medicine DallasTX United States Internal Medicine, University of Texas Southwestern Medical Center, Dallas, TX, United States;; 3 University of Texas Southwestern Medical Center DallasTX United States Radiology, University of Texas Southwestern Medical Center, Dallas, TX, United States

**Keywords:** Cakut [Supplementary Concept], Salvage Therapy, Kidney Function Tests

## Abstract

**Purpose:**

We aimed to assess failure rates of salvage interventions and changes in split kidney function (SKF) following failed primary repair of ureteropelvic junction obstruction (UPJO).

**Materials and Methods:**

A retrospective review of adult patients at an academic medical center who underwent salvage intervention following primary treatment for UPJO was performed. Symptomatic failure was defined as significant flank pain. Radiographic failure was defined as no improvement in drainage or a decrease in SKF by ≥7%. Overall failure, the primary outcome, was defined as symptomatic failure, radiographic failure, or both.

**Results:**

Between 2008-2017, 34 patients (median age 38 years, 50% men) met study criteria. UPJO management was primary pyeloplasty/secondary endopyelotomy for 21/34 (62%), primary pyeloplasty/secondary pyeloplasty for 6/34 (18%), and primary endopyelotomy/secondary pyeloplasty for 7/34 (21%). Median follow-up was 3.3 years following secondary intervention. Patients undergoing primary pyeloplasty/secondary endopyelotomy had significantly higher overall failure than those undergoing primary pyeloplasty/secondary pyeloplasty (16/21 [76%] vs. 1/6 [17%], p=0.015). Among patients undergoing secondary endopyelotomy, presence of a stricture on retrograde pyelogram, stricture length, and SKF were not associated with symptomatic, radiographic, or overall failure. Serial renography was performed for 28/34 (82%) patients and 2/28 (7%) had a significant decline in SKF.

**Conclusions:**

Following failed primary pyeloplasty, secondary endopyelotomy had a greater overall failure rate than secondary pyeloplasty. No radiographic features assessed were associated with secondary endopyelotomy failure. Secondary intervention overall failure rates were higher than reported in the literature. Unique to this study, serial renography demonstrated that significant functional loss was overall infrequent.

## INTRODUCTION

Pyeloplasty is the gold standard for the initial repair of ureteropelvic junction obstruction (UPJO), and most surgical series have demonstrated a low failure rate with this approach (≤10%) (1). Nonetheless, failed primary intervention presents a significant challenge. The most commonly utilized secondary interventions in this setting are endopyelotomy and pyeloplasty (1). Anatomical complexities sometimes necessitate other techniques such as ureterocalicostomy (2), buccal ureteroplasty (3), bowel interposition (4), and autotransplant (5), among others.

Series comparing endopyelotomy and pyeloplasty following failed primary pyeloplasty demonstrated failure rates of 29-62% and 0-13%, respectively (6–8). Failure definitions included persistent symptoms, lack of radiographic improvement, and need for further surgery. Different failure definitions and varied follow-up protocols are among the factors complicating the interpretation of head-to-head comparisons of endopyelotomy and pyeloplasty as secondary interventions.

Based on our experience managing UPJO in the salvage setting, we hypothesized that, following primary pyeloplasty, the overall failure rate of secondary endopyelotomy significantly exceeded that of secondary pyeloplasty, and that both exceeded failure rates previously reported in the literature. Beyond testing this primary hypothesis, we also aimed to assess radiographic features associated with secondary endopyelotomy failure. Finally, unique to this study, we evaluated changes in split kidney function (SKF) over time among patients failing primary intervention.

## MATERIALS AND METHODS

### Patient population

Following Institutional Review Board approval (ID#: STU 102017-002), we performed a retrospective review of all adult patients at an academic tertiary care center who underwent salvage intervention for UPJO following failed primary intervention between 2008-2017. Patients who failed primary intervention performed at our institution, as well as outside institutions, meeting definitions of symptomatic failure or radiographic failure were included (see “Outcome assessment” for definitions). Patients without at least one assessment for flank pain and one radiographic evaluation following postoperative ureteral stent removal were excluded. We also excluded patients managed with buccal ureteroplasty due to limited experience with this technique during the study period. Finally, patients with a history of upper urinary tract reconstruction unrelated to UPJO were excluded due to the possibility of impaired drainage not attributable to UPJO.

### Intervention selection

The choice of salvage intervention was determined based on a shared decision-making process. Informed consent was obtained before all procedures. Secondary endopyelotomy was preferred following failed primary pyeloplasty when stricture length was ≤2cm, there was no evidence of a crossing vessel, there was mild or moderate hydronephrosis, and ipsilateral SKF was >25%. Secondary pyeloplasty was recommended following failed primary pyeloplasty in the absence of one or more of these favorable factors. Secondary pyeloplasty was also preferred for salvage following failed primary endopyelotomy. If tertiary intervention was pursued, the failed secondary intervention was not repeated. Ureterocalicostomy was recommended when endopyelotomy had already failed and excretory imaging or retrograde pyelography demonstrated inadequate renal pelvis tissue, precluding pyeloplasty. Finally, nephrectomy was usually advised for symptomatic patients with ipsilateral SKF <20%, or if further salvage intervention was deemed futile through shared decision-making. Salvage interventions were performed without a ureteral stent in place during the weeks preceding the procedure, patients requiring drainage before salvage interventions underwent nephrostomy placement. Urine cultures were obtained prior to each intervention and positive results were appropriately treated with antibiotics.

### Surgical techniques

All endopyelotomies were performed retrograde using a flexible ureteroscope and Holmium laser fiber, following a retrograde pyelogram for anatomical reassessment including stricture length measurement. A posterolateral transmural incision was made. Calibration was then performed using a ureteral dilating balloon under fluoroscopy. A dual diameter endopyelotomy stent typically remained in place for 4-6 weeks. Patients were either discharged the same day or admitted for overnight observation. The urethral catheter, if placed, was typically removed the day after the procedure. Three endourologists at our institution performed the endopyelotomies in this series.

Prior to pyeloplasty at our institution, intravenous, retrograde, antegrade, computed tomography (CT), or magnetic resonance urography was performed to evaluate stricture length. Open, laparoscopic, and robotic approaches were utilized for pyeloplasty (Supplementary Table-S1). Techniques including Anderson-Hynes, Heineke-Mikulicz, and spiral flap repairs were used depending on anatomic presentation and surgeon preference. A ureteral stent was usually placed in an antegrade fashion during the procedure and removed 4-6 weeks later. A closed suction drain was placed and removed prior to discharge if there was no suspicion of urine leak. The urethral catheter was typically removed on the day after the procedure. Stent, drain, and catheter management was similar for ureterocalicostomies, all of which were performed open. One endourologist and one reconstructive urologist at our institution performed the pyeloplasties and ureterocalicostomies in this series.

### Outcome assessment

Symptomatic failure was defined as significant flank pain following intervention. All patients had at least one outpatient encounter following ureteral stent removal in which an assessment for symptomatic failure was made. The date of last follow-up was the last documented in-person or telephone encounter regarding UPJO. A post-operative radiographic assessment was planned for 4-6 weeks following ureteral stent removal. Radiographic failure was defined as no improvement in drainage, i.e., no interval decrease in a baseline abnormal t½ (any t½ >10 minutes was considered abnormal), or as an interval decrease in SKF by ≥7% as assessed on mercaptoacetyltriglycine (MAG3) diuretic renography (9). When renography was not performed, radiologist and/or surgeon interpretation of lack of improvement in drainage on intravenous, retrograde, antegrade, CT, or magnetic resonance urography was used to define radiographic failure. Overall failure, the primary outcome of interest in this study, was defined as symptomatic failure, radiographic failure, or both. Aside from symptomatic failure and radiographic failure, additional secondary outcomes of interest included tertiary intervention and nephrectomy. Complications were graded according to the Clavien-Dindo classification (10).

### Statistical Analysis

Patients were categorized based on the sequence of interventions: primary pyeloplasty/secondary endopyelotomy, primary pyeloplasty/secondary pyeloplasty, and primary endopyelotomy/secondary pyeloplasty. Differences in the primary and secondary outcomes were assessed for the primary pyeloplasty/secondary endopyelotomy and primary pyeloplasty/secondary pyeloplasty groups, but not for the primary endopyelotomy/secondary pyeloplasty group, as pyeloplasty is preferred over endopyelotomy in the primary setting. Patients undergoing primary endopyelotomy/secondary pyeloplasty were still included in the overall cohort for measurement of SKF over time.

Median follow-up differences between groups were assessed using the Mann-Whitney U test. Differences in baseline characteristics and perioperative outcomes were assessed using Fisher’s exact test for categorical variables and the Mann-Whitney U test for continuous variables. Differences in the primary and secondary outcomes of interest were assessed using Fisher’s exact test. Differences in radiographic findings preceding secondary endopyelotomy were assessed using Fisher’s exact test for categorical variables (presence of stricture) and the Mann-Whitney U test for continuous variables (stricture length and SKF).

The primary outcome (overall failure) is a composite of two other outcomes (symptomatic failure and radiographic failure); thus, to account for multiple hypothesis testing, a Bonferroni correction was applied, dividing the standard statistical significance threshold (p <0.05) by 3, making p < statistically significant in the assessment of these 3 failure outcomes. Statistical significance was otherwise defined as p <0.05. All p values were two-sided. Statistical analyses were performed using MATLAB (The MathWorks, Inc., Natick, MA, USA).

## RESULTS

### Patient population

**Figure 1:**
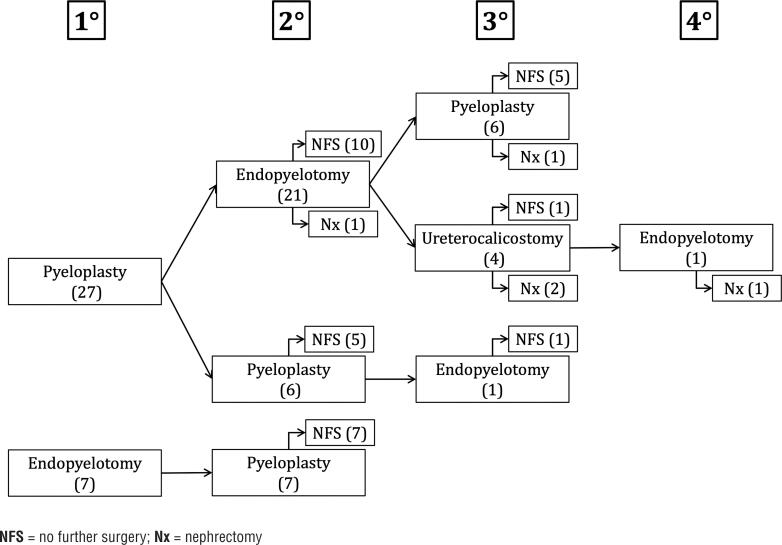
Sequence of primary (1°), secondary (2°), tertiary (3°), and quaternary (4°) interventions.

We identified 34 adult patients (median age 38 years [range 19-82]; 17 [50%] men) meeting study criteria who underwent salvage intervention between 2008-2017 among >200 adult patients treated for UPJO at our institution, in addition to outside referrals. Four patients had been excluded: 2 without available post-operative imaging, 1 who underwent buccal ureteroplasty, and 1 with a history of ureteral reimplantation. Baseline characteristics and perioperative outcomes are summarized in Tables 1A and 1B, respectively. There were no statistically significant differences in age, body mass index (BMI), or American Society of Anesthesiologists (ASA) score between the primary pyeloplasty/secondary endopyelotomy and primary pyeloplasty/secondary pyeloplasty groups. In the setting of the secondary intervention, patients undergoing primary pyeloplasty/secondary endopyelotomy had significantly shorter operative time and length of stay, as well as lower estimated blood loss. The detailed sequence of interventions is illustrated in Figure-1.

**Table 1:** (A) Baseline characteristics, (B) perioperative outcomes, and (C) long-term outcomes of patients undergoing salvage intervention for ureteropelvic junction obstruction stratified by primary (1°) and secondary (2°) interventions.

			All patients (n=34)	1° pyeloplasty, 2° endopyelotomy (n=21, 62%)	1° pyeloplasty, 2° pyeloplasty (n=6, 18%)	1° endopyelotomy, 2° pyeloplasty (n=7, 21%)	p value*
Table 1-A) **Baseline characteristics**							
	Median age, years (range)		38 (19-82)	36 (21-79)	43 (19-61)	40 (30-82)	0.5
**Gender**							
	Female		17 (50%)	10 (48%)	2 (33%)	5 (71%)	0.7
	Male		17 (50%)	11 (52%)	4 (67%)	2 (29%)	
**Race**†							
	White		26 (81%)	14 (70%)	6 (100%)	6 (100%)	0.3
	Black		4 (13%)	4 (20%)	0 (0%)	0 (0%)	
	Asian		2 (6%)	2 (10%)	0 (0%)	0 (0%)	
**Ethnicity**‡							
	Non-Hispanic		29 (91%)	17 (85%)	6 (100%)	6 (100%)	1.0
	Hispanic		3 (9%)	3 (15%)	0 (0%)	0 (0%)	
**Side**							
	Left		17 (50%)	13 (62%)	3 (50%)	1 (14%)	0.7
	Right		17 (50%)	8 (38%)	3 (50%)	6 (86%)	
	Median BMI, kg/m^2^ (range)		28 (20-45)	28 (20-36)	28 (22-45)	24 (21-37)	0.8
	Median ASA score (range)		2 (1-3)	2 (1-3)	2 (1-3)	2 (1-3)	0.5
	History of urolithiasis		15 (44%)	9 (43%)	3 (50%)	3 (43%)	1.0
	History of UTIs		13 (38%)	8 (38%)	3 (50%)	2 (29%)	0.7
Table 1-B) **Perioperative outcomes**§							
	Median OR time, min (range)		109 (50-343)	74 (50-131)	187 (131-279)	270 (137-343)	0.001
	Median LOS, days (range)		1 (0-7)	0 (0-2)	2 (2-7)	2 (2-3)	<0.001
	Median EBL, mL (range)		5 (0-150)	2 (0-50)	25 (10-150)	50 (10-50)	0.01
Table 1-C) **Long-term outcomes**							
	**Failure**§,++						
		Symptomatic	14 (41%)	12 (57%)	0 (0%)	2 (29%)	0.020
		Radiographic	12 (35%)	10 (48%)	1 (17%)	1 (14%)	0.3
		Overall	20 (59%)	16 (76%)	1 (17%)	3 (43%)	0.015
	Tertiary intervention		11 (32%)	10 (48%)	1 (17%)	0 (0%)	0.3
	Nephrectomy		5 (15%)	5 (24%)	0 (0%)	0 (0%)	0.6

**BMI =** body mass index; **ASA =** American Society of Anesthesiologists; **UTI =** urinary tract infection; **OR =** operating room; **EBL =**estimated blood loss; **LOS =** length of stay.

* Comparison of primary pyeloplasty/secondary endopyelotomy and primary pyeloplasty/secondary pyeloplasty groups;

† p value reflects comparison of non-white and white race; race data available for 32/34 (94%) patients;

‡ Ethnicity data available for 32/34 (94%) patients;

§ Pertains to the secondary intervention.

++ Statistical significance defined as p< 0.16 or failure outcomes.

### Follow-up

Median follow-up was 3.3 years (interquartile range [IQR] 1.4-6.5) after the secondary intervention across the entire cohort. Median follow-up after the secondary intervention was 3.3 years among patients undergoing primary pyeloplasty/secondary endopyelotomy, compared to 6.9 years among patients undergoing primary pyeloplasty/secondary pyeloplasty (p=0.06); patients undergoing primary endopyelotomy/secondary pyeloplasty had the shortest follow-up (median 1.7 years) but this was not statistically significantly less than the other groups (p=0.2 and p=0.8, respectively). Median time from primary to secondary intervention was 1.3 years (IQR 0.5-7.9) and median time from primary to tertiary intervention was 1.7 years (IQR 1.0-8.6).

### Secondary intervention outcomes

Long-term outcomes are summarized in Table-1C. Compared to primary pyeloplasty/secondary pyeloplasty, patients who underwent primary pyeloplasty/secondary endopyelotomy had significantly higher overall failure (p=0.015). There were no statistically significant differences in symptomatic failure, radiographic failure, need for tertiary procedure, or need for nephrectomy between these two groups. Complications following salvage interventions are listed in Supplementary Table-S2.

### Radiographic features preceding secondary endopyelotomy

Findings on retrograde pyelogram and diuretic renography prior to secondary endopyelotomy are shown in Supplementary Table-S3. Presence of a discernable stricture on retrograde pyelogram was not associated with failure of any type. Stricture length on retrograde pyelogram as well as SKF were not statistically significantly different for patients with versus without failure of any type.

### Change in split kidney function

**Figure 2:**
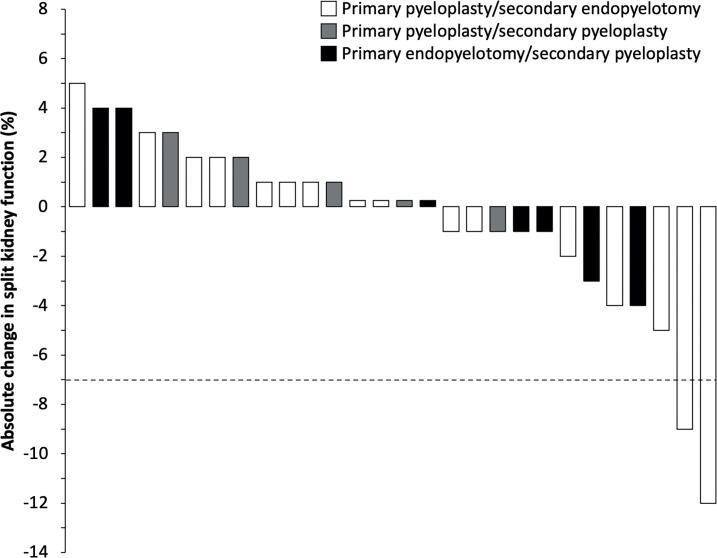
Waterfall plot of interval change in split kidney function for 28 patients who underwent diuretic renography both before and after salvage interventions. The dashed line at -7% corresponds to the selected definition of a significant decrease in split kidney function.

A majority (28/34 [82%]) of the cohort underwent diuretic renography both before and after salvage interventions; the interval changes in SKF are displayed in Figure-2. Among these patients, the median time spanned by renography studies was 2.4 years (IQR 0.9-5.6). The median baseline SKF was 45% (range 25% to 63%), and the median absolute change in SKF over this period was 0% (range-12% to +5%). Two (7%) patients developed a significant decline in SKF, defined as a decrease ≥7% (9), both underwent primary pyeloplasty, secondary endopyelotomy, and tertiary pyeloplasty.

## DISCUSSION

The primary hypothesis of this study was that, following primary pyeloplasty, the overall failure rate of secondary endopyelotomy exceeded that of secondary pyeloplasty, and that both exceeded failure rates previously reported in the literature. Secondary endopyelotomy did have a significantly higher overall failure rate compared to secondary pyeloplasty. Furthermore, overall failure rates for both interventions following primary pyeloplasty were indeed higher than reported in other series: 76% for secondary endopyelotomy and 17% for secondary pyeloplasty. All patients experiencing significant functional loss (n=2) and nephrectomy (n=5) did so after primary pyeloplasty/secondary endopyelotomy. This suggests that secondary endopyelotomy may be associated with adverse outcomes, including kidney loss.

Established risk factors for endopyelotomy failure from series containing mostly primary cases include stricture length, degree of hydronephrosis, SKF, and crossing vessels (11). In a series of patients undergoing endopyelotomy after failed pyeloplasty with a 12.5% failure rate, Jabbour et al. proposed that massive hydronephrosis and low SKF were risk factors for failure (12). We also evaluated several factors in the context of secondary endopyelotomy failure, namely presence and length of stricture on retrograde pyelogram immediately preceding laser incision, as well as preoperative SKF. None of these variables was associated with failure. Assessments of hydronephrosis severity were not performed due to the presence of nephrostomies decompressing the collecting system for several patients. Patients with known crossing vessels were not offered endopyelotomy, though no imaging assessment of this was consistently utilized (e.g., CT angiography). Given that stricture characteristics (≤2cm for all) and SKF (>25% for all) were not associated with secondary endopyelotomy outcomes in this series, we conclude that even well-selected endopyelotomy candidates have a significant risk of failure.

To our knowledge, this is the first study of salvage interventions for UPJO to evaluate the change in SKF over time. Despite the frequency of failed salvage intervention, only two patients had a significant decline in SKF, emphasizing the predominance of flank pain and impaired drainage as the reason for failure, not functional loss. This is consistent with the finding that symptomatic failure was more common than radiographic failure following salvage interventions in this series.

Pyeloplasty is favored over endopyelotomy in the primary setting based on long-term data, including those of Dimarco et al. who found that ten-year recurrence-free survival after primary pyeloplasty and primary endopyelotomy were 75% and 41%, respectively (13). Our series and several other retrospective head-to-head comparisons following failed primary pyeloplasty (6–8) suggest a similar dichotomy in the secondary setting, with superior outcomes of pyeloplasty (failure 0-17%) compared to endopyelotomy (failure 29-76%). These data in the context of decreased morbidity and increased availability of laparoscopic/robotic approaches (14, 15) make secondary pyeloplasty an increasingly attractive approach. Indeed, one study comparing redo laparoscopic pyeloplasty to primary laparoscopic pyeloplasty in a matched fashion found no evident differences in complications or outcomes, with the exception of operative time (16). We were unable to compare outcomes of open, laparoscopic, and robotic salvage pyeloplasty in the present study due to limited sample size.

Data favoring outcomes of secondary pyeloplasty over those of secondary endopyelotomy do not necessarily render secondary endopyelotomy a procedure without merit. Complications following endopyelotomy are infrequent and usually low grade, length of hospital stay following endopyelotomy is consistently shorter than it is for pyeloplasty (17). Therefore, it may be the preferred option for patients with significant medical comorbidities. However, in our series, no significant baseline differences in age, BMI, or ASA score were detected between patients undergoing primary pyeloplasty/secondary endopyelotomy and primary pyeloplasty/secondary pyeloplasty.

Pursuit of salvage intervention for UPJO is made through a shared decision-making process based on the best available evidence. Selection bias is a major obstacle in retrospective studies comparing these procedures. Patients undergoing secondary endopyelotomy were well-selected based on radiographic findings, potentially generating a bias favoring patients undergoing secondary endopyelotomy over those undergoing secondary pyeloplasty, the direction of this bias may explain why the secondary pyeloplasty failure rate was higher than previously reported. Only a randomized trial can overcome this selection bias inherent in our cohort and others. The feasibility of such a trial is limited by the low incidence of UPJO and the low failure rate of primary pyeloplasty. A prospective multi-institutional registry or a meta-analysis of available retrospective data may be practical avenues to further assess outcomes of salvage intervention for UPJO.

A number of innovations have the potential to improve management of patients requiring salvage intervention for UPJO, though they were beyond the scope of this study. Robotic buccal ureteroplasty is a newer technique which was excluded from this series due to limited experience at our center (n=1), though it should be considered in salvage management of UPJO given low reported failure rates (3). Other techniques such as augmentation with cryopreserved placental tissue also have the potential to increase the probability of success (18). Additionally, the extent to which histologic features of UPJ specimens (19) or renal parenchymal biopsies (20) are associated with outcomes of salvage intervention for UPJO was not assessed in this study, and this may be an area for future research.

Strengths of this study include strict exclusion criteria, stringent failure definitions, a standardized, thorough technique for endopyelotomy, median follow-up of over three years after secondary intervention, evaluation of radiographic features pertinent to secondary endopyelotomy failure, and serial renography studies performed for the vast majority of patients to assess changes in SKF. Study limitations include sources of heterogeneity such as primary interventions performed at outside institutions for most patients, variation in pyeloplasty approach/technique, and potential inter-rater variability in imaging interpretation. The sample size was limited, particularly for patients undergoing secondary pyeloplasty following failed primary pyeloplasty. A larger cohort could yield additional detectable differences between groups in outcomes such as symptomatic failure, radiographic failure, need for tertiary intervention, and need for nephrectomy, as well as further elucidate risk factors for adverse outcomes and perioperative management strategies such as the optimal ureteral stent duration. Finally, selection bias is present in all retrospective comparisons of endopyelotomy and pyeloplasty, including this study.

## CONCLUSIONS

In this series of patients undergoing salvage intervention for UPJO, following failed primary pyeloplasty, secondary endopyelotomy had a significantly higher overall failure rate compared to secondary pyeloplasty. Failure rates of salvage interventions were uniformly higher in this study than previously reported. No radiographic features assessed were found to be associated with secondary endopyelotomy failure. Unique to this work, serial diuretic renography studies demonstrated that significant loss of function was overall infrequent.

## ABBREVIATIONS AND ACRONYMS

ASA: American Society of Anesthesiologists
BMI: body mass index
CT: computed tomography
IQR: interquartile range
MAG3: mercaptoacetyltriglycine
SKF: split kidney function
UPJO: ureteropelvic junction obstruction

## APPENDIX

**Supplementary Table S1:** Pyeloplasty operative approach.

	All pyeloplasties	1° pyeloplasties	2° pyeloplasties	3° pyeloplasties
Open	13 (28%)	8 (30%)	3 (23%)	2 (33%)
Laparoscopic	15 (33%)	9 (33%)	5 (38%)	1 (17%)
Robotic	18 (39%)	10 (37%)	5 (38%)	3 (50%)
**Total**	**46**	**27**	**13**	**6**

1° primary

2° secondary

3° tertiary

**Supplementary Table S2:** Surgical complications following salvage intervention for ureteropelvic junction obstruction.

Surgical intervention	Complication	Grade
Tertiary ureterocalicostomy	Wound infection	I
Secondary endopyelotomy	Pyelonephritis	II
Tertiary robotic pyeloplasty	Pyelonephritis	II
Tertiary ureterocalicostomy	Pneumonia, parapneumonic effusion requiring drainage	IIIa
Secondary open pyeloplasty	Pulmonary embolism, rhabdomyolysis requiring intensive care	IV

**Supplementary Table S3:** Radiographic findings preceding secondary endopyelotomy; patients are stratified by failure type.

	All secondary endopyelotomies (n=21)	Symptomatic failure		Radiographic failure		Overall failure	
		Yes	No	Yes	No	Yes	No
		(n=12, 57%)	(n=9, 43%)	(n=10, 48%)	(n=11, 52%)	(n=16, 76%)	(n=5, 24%)
Stricture present on RGPG*	13 (68%)	5 (50%)	8 (89%)	7 (88%)	6 (55%)	9 (64%)	4 (80%)
		p=0.14		p=0.2		p=1.0	
Median stricture length, cm (range)*	1.0 (0.5-2.0)	1.0 (0.5-2.0)	1.0 (0.5-1.5)	1.0 (0.5-1.5)	1.0 (0.5-2.0)	1.0 (0.5-2.0)	1.0 (0.5-1.0)
		p=0.8		p=0.3		p=0.9	
Median SKF, % (range)†	44 (26-63)	46 (30-63)	43 (26-54)	42 (26-63)	45 (29-54)	44 (26-63)	44 (29-54)
		p=0.6		p=0.7		p=0.7	

**RGPG** = retrograde pyelogram; **SKF** = split kidney function.

* **RGPG =** performed at time of secondary endopyelotomy available for 19/21 (90%) patients.

† **SKF** values prior to secondary endopyelotomy available for 20/21 (95%) patients.
